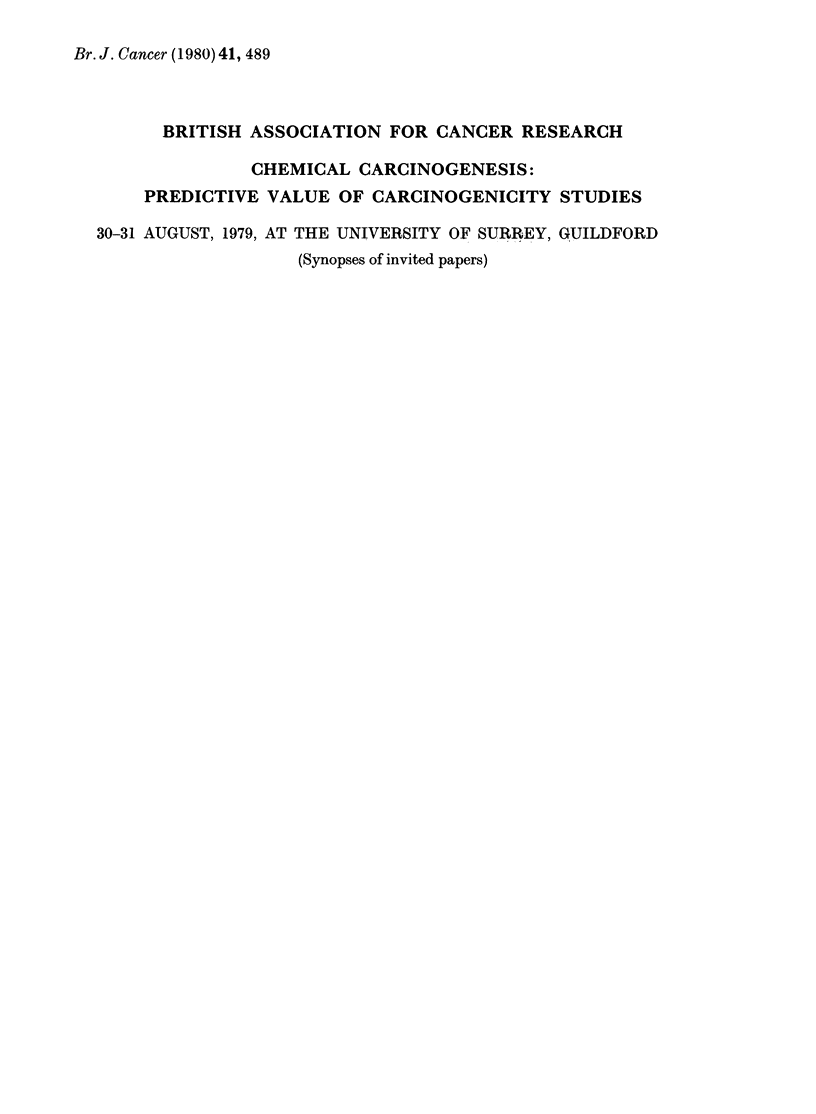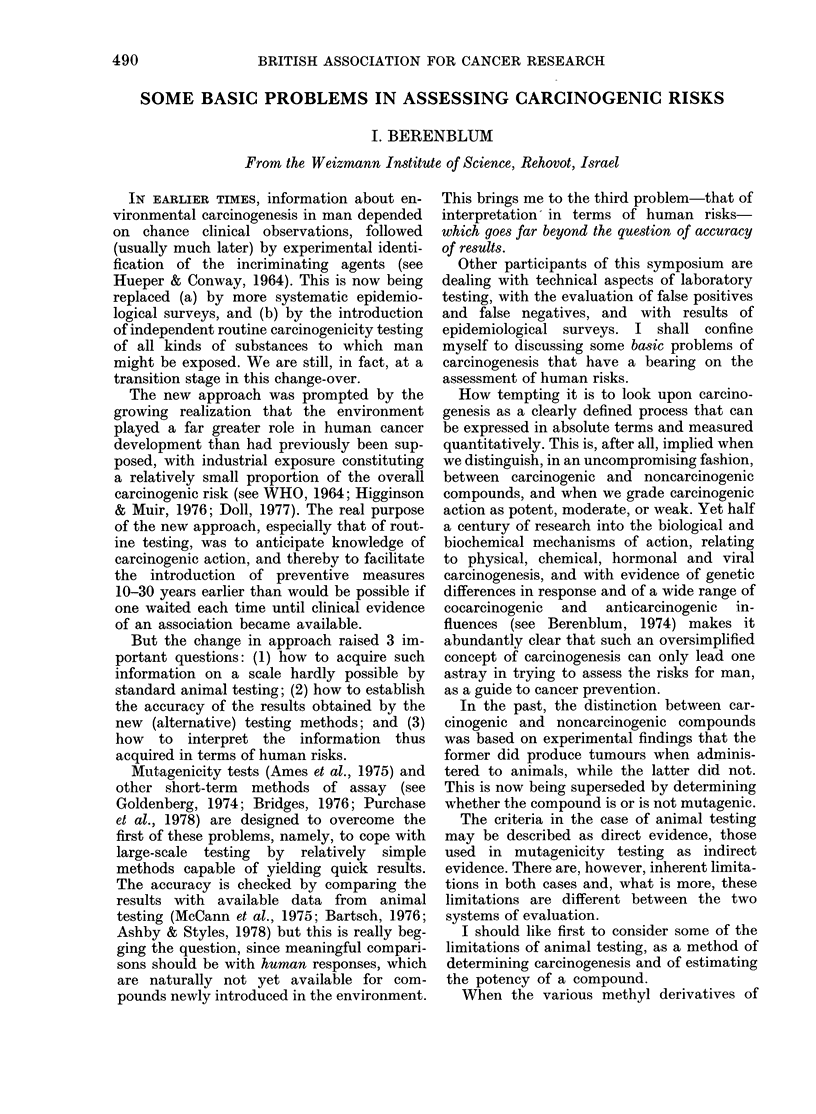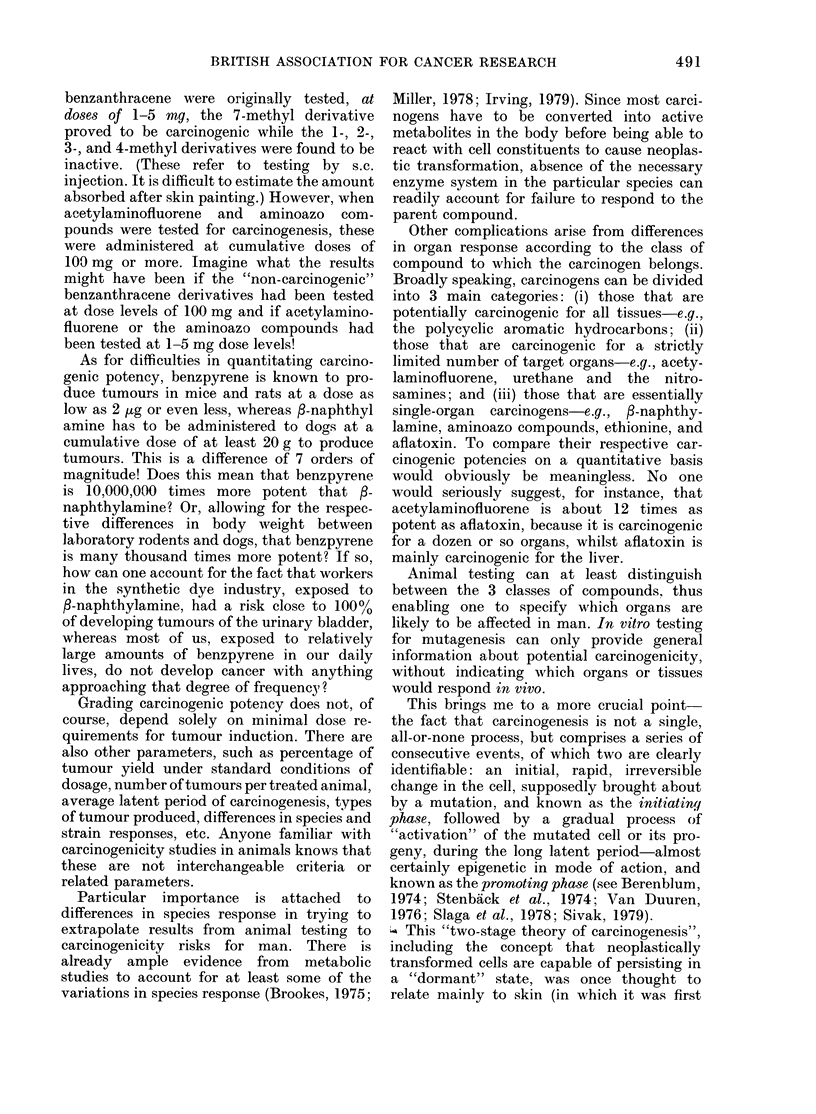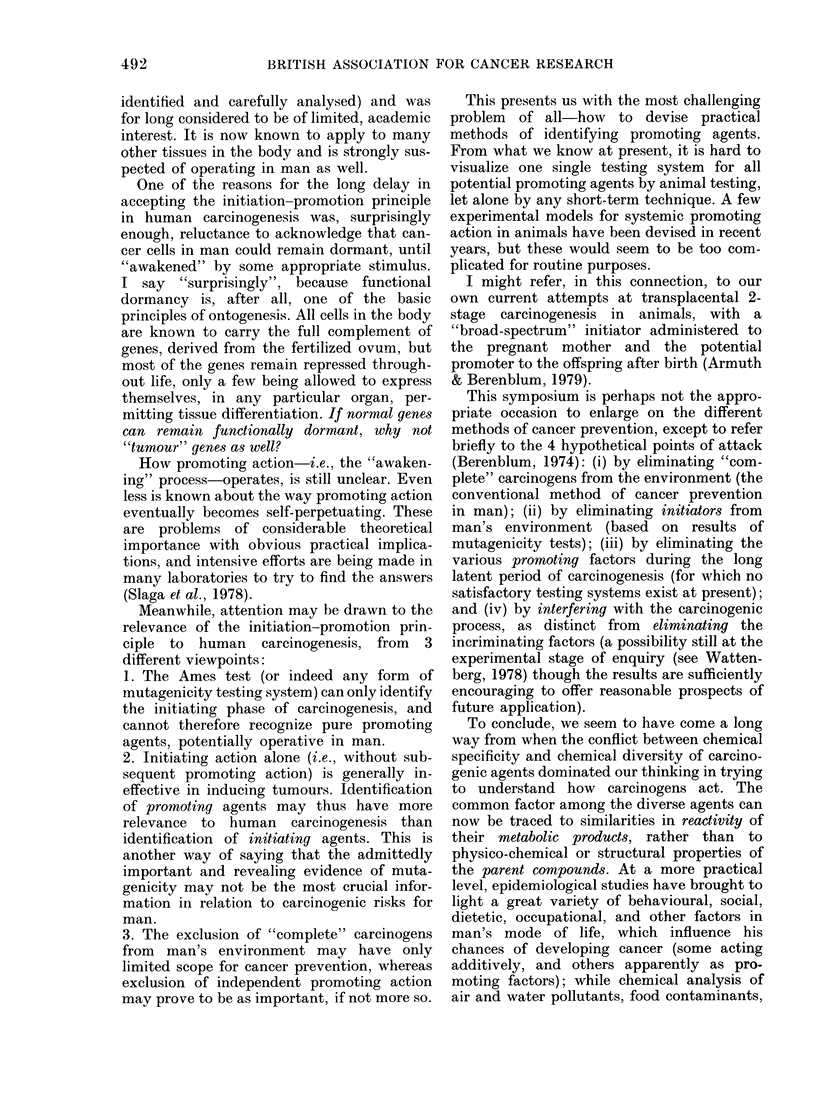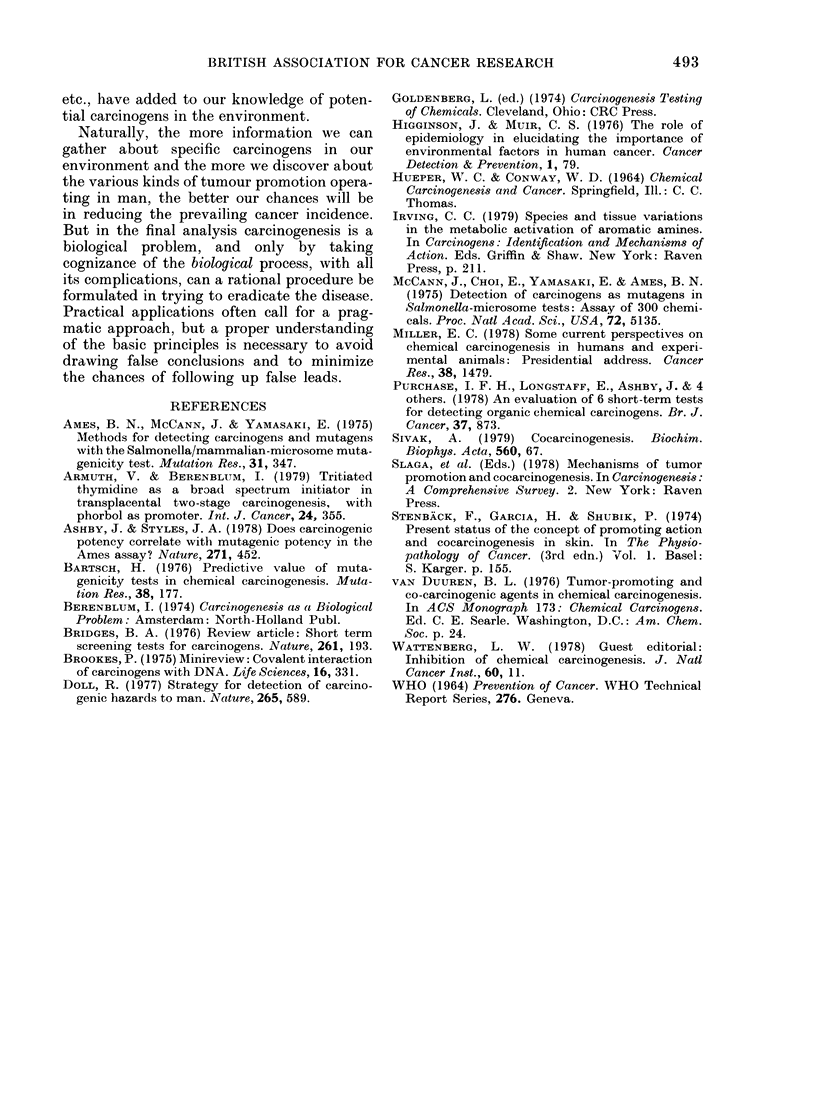# Some basic problems in assessing carcinogenic risks.

**DOI:** 10.1038/bjc.1980.76

**Published:** 1980-03

**Authors:** I. Berenblum


*Br. J. Cancer* (1980) **41**, 489

### BRITISH ASSOCIATION FOR CANCER RESEARCH

### CHEMICAL CARCINOGENESIS: PREDICTIVE VALUE OF CARCINOGENICITY STUDIES

30–31 AUGUST, 1979, AT THE UNIVERSITY OF SURREY, GUILDFORD

(Synopses of invited papers)

### SOME BASIC PROBLEMS IN ASSESSING CARCINOGENIC RISKS

I. BERENBLUM

*From the Weizmann Institute of Science, Rehovot, Israel*

IN EARLIER TIMES, information about environmental carcinogenesis in man depended on chance clinical observations, followed (usually much later) by experimental identification of the incriminating agents (see Hueper & Conway, 1964). This is now being replaced (a) by more systematic epidemiological surveys, and (b) by the introduction of independent routine carcinogenicity testing of all kinds of substances to which man might be exposed. We are still, in fact, at a transition stage in this change-over.

The new approach was prompted by the growing realization that the environment played a far greater role in human cancer development than had previously been supposed, with industrial exposure constituting a relatively small proportion of the overall carcinogenic risk (see WHO, 1964; Higginson & Muir, 1976; Doll, 1977). The real purpose of the new approach, especially that of routine testing, was to anticipate knowledge of carcinogenic action, and thereby to facilitate the introduction of preventive measures 10–30 years earlier than would be possible if one waited each time until clinical evidence of an association became available.

But the change in approach raised 3 important questions: (1) how to acquire such information on a scale hardly possible by standard animal testing; (2) how to establish the accuracy of the results obtained by the new (alternative) testing methods; and (3) how to interpret the information thus acquired in terms of human risks.

Mutagenicity tests (Ames *et al.*, 1975) and other short-term methods of assay (see Goldenberg, 1974; Bridges, 1976; Purchase *et al.*, 1978) are designed to overcome the first of these problems, namely, to cope with large-scale testing by relatively simple methods capable of yielding quick results. The accuracy is checked by comparing the results with available data from animal testing (McCann *et al.*, 1975; Bartsch, 1976; Ashby & Styles, 1978) but this is really begging the question, since meaningful comparisons should be with *human* responses, which are naturally not yet available for compounds newly introduced in the environment. This brings me to the third problem—that of interpretation in terms of human risks—*which goes far beyond the question of accuracy of results*.

Other participants of this symposium are dealing with technical aspects of laboratory testing, with the evaluation of false positives and false negatives, and with results of epidemiological surveys. I shall confine myself to discussing some *basic* problems of carcinogenesis that have a bearing on the assessment of human risks.

How tempting it is to look upon carcinogenesis as a clearly defined process that can be expressed in absolute terms and measured quantitatively. This is, after all, implied when we distinguish, in an uncompromising fashion, between carcinogenic and noncarcinogenic compounds, and when we grade carcinogenic action as potent, moderate, or weak. Yet half a century of research into the biological and biochemical mechanisms of action, relating to physical, chemical, hormonal and viral carcinogenesis, and with evidence of genetic differences in response and of a wide range of cocarcinogenic and anticarcinogenic influences (see Berenblum, 1974) makes it abundantly clear that such an oversimplified concept of carcinogenesis can only lead one astray in trying to assess the risks for man, as a guide to cancer prevention.

In the past, the distinction between carcinogenic and noncarcinogenic compounds was based on experimental findings that the former did produce tumours when administered to animals, while the latter did not. This is now being superseded by determining whether the compound is or is not mutagenic.

The criteria in the case of animal testing may be described as direct evidence, those used in mutagenicity testing as indirect evidence. There are, however, inherent limitations in both cases and, what is more, these limitations are different between the two systems of evaluation.

I should like first to consider some of the limitations of animal testing, as a method of determining carcinogenesis and of estimating the potency of a compound.

When the various methyl derivatives of benzanthracene were originally tested, *at doses of* 1–5 *mg*, the 7-methyl derivative proved to be carcinogenic while the 1-, 2-, 3-, and 4-methyl derivatives were found to be inactive. (These refer to testing by s.c. injection. It is difficult to estimate the amount absorbed after skin painting.) However, when acetylaminofluorene and aminoazo compounds were tested for carcinogenesis, these were administered at cumulative doses of 100 mg or more. Imagine what the results might have been if the "non-carcinogenic" benzanthracene derivatives had been tested at dose levels of 100 mg and if acetylaminofluorene or the aminoazo compounds had been tested at 1–5 mg dose levels!

As for difficulties in quantitating carcinogenic potency, benzpyrene is known to produce tumours in mice and rats at a dose as low as 2 μg or even less, whereas β-naphthyl amine has to be administered to dogs at a cumulative dose of at least 20 g to produce tumours. This is a difference of 7 orders of magnitude! Does this mean that benzpyrene is 10,000,000 times more potent that β-naphthylamine? Or, allowing for the respective differences in body weight between laboratory rodents and dogs, that benzpyrene is many thousand times more potent? If so, how can one account for the fact that workers in the synthetic dye industry, exposed to β-naphthylamine, had a risk close to 100% of developing tumours of the urinary bladder, whereas most of us, exposed to relatively large amounts of benzpyrene in our daily lives, do not develop cancer with anything approaching that degree of frequency?

Grading carcinogenic potency does not, of course, depend solely on minimal dose requirements for tumour induction. There are also other parameters, such as percentage of tumour yield under standard conditions of dosage, number of tumours per treated animal, average latent period of carcinogenesis, types of tumour produced, differences in species and strain responses, etc. Anyone familiar with carcinogenicity studies in animals knows that these are not interchangeable criteria or related parameters.

Particular importance is attached to differences in species response in trying to extrapolate results from animal testing to carcinogenicity risks for man. There is already ample evidence from metabolic studies to account for at least some of the variations in species response (Brookes, 1975; Miller, 1978; Irving, 1979). Since most carcinogens have to be converted into active metabolites in the body before being able to react with cell constituents to cause neoplastic transformation, absence of the necessary enzyme system in the particular species can readily account for failure to respond to the parent compound.

Other complications arise from differences in organ response according to the class of compound to which the carcinogen belongs. Broadly speaking, carcinogens can be divided into 3 main categories: (i) those that are potentially carcinogenic for all tissues—*e.g.*, the polycyclic aromatic hydrocarbons; (ii) those that are carcinogenic for a strictly limited number of target organs—*e.g.*, acetylaminofluorene, urethane and the nitrosamines; and (iii) those that are essentially single-organ carcinogens—*e.g.*, β-naphthylamine, aminoazo compounds, ethionine, and aflatoxin. To compare their respective carcinogenic potencies on a quantitative basis would obviously be meaningless. No one would seriously suggest, for instance, that acetylaminofluorene is about 12 times as potent as aflatoxin, because it is carcinogenic for a dozen or so organs, whilst aflatoxin is mainly carcinogenic for the liver.

Animal testing can at least distinguish between the 3 classes of compounds, thus enabling one to specify which organs are likely to be affected in man. *In vitro* testing for mutagenesis can only provide general information about potential carcinogenicity, without indicating which organs or tissues would respond *in vivo*.

This brings me to a more crucial point—the fact that carcinogenesis is not a single, all-or-none process, but comprises a series of consecutive events, of which two are clearly identifiable: an initial, rapid, irreversible change in the cell, supposedly brought about by a mutation, and known as the *initiating phase*, followed by a gradual process of "activation" of the mutated cell or its progeny, during the long latent period—almost certainly epigenetic in mode of action, and known as the *promoting phase* (see Berenblum, 1974; Stenbäck *et al.*, 1974; Van Duuren, 1976; Slaga *et al.*, 1978; Sivak, 1979).

This "two-stage theory of carcinogenesis", including the concept that neoplastically transformed cells are capable of persisting in a "dormant" state, was once thought to relate mainly to skin (in which it was first identified and carefully analysed) and was for long considered to be of limited, academic interest. It is now known to apply to many other tissues in the body and is strongly suspected of operating in man as well.

One of the reasons for the long delay in accepting the initiation–promotion principle in human carcinogenesis was, surprisingly enough, reluctance to acknowledge that cancer cells in man could remain dormant, until "awakened" by some appropriate stimulus. I say "surprisingly", because functional dormancy is, after all, one of the basic principles of ontogenesis. All cells in the body are known to carry the full complement of genes, derived from the fertilized ovum, but most of the genes remain repressed throughout life, only a few being allowed to express themselves, in any particular organ, permitting tissue differentiation. *If normal genes can remain functionally dormant, why not "tumour" genes as well?*

How promoting action—*i.e.*, the "awakening" process—operates, is still unclear. Even less is known about the way promoting action eventually becomes self-perpetuating. These are problems of considerable theoretical importance with obvious practical implications, and intensive efforts are being made in many laboratories to try to find the answers (Slaga *et al.*, 1978).

Meanwhile, attention may be drawn to the relevance of the initiation–promotion principle to human carcinogenesis, from 3 different viewpoints:

1. The Ames test (or indeed any form of mutagenicity testing system) can only identify the initiating phase of carcinogenesis, and cannot therefore recognize pure promoting agents, potentially operative in man.
2. Initiating action alone (*i.e.*, without subsequent promoting action) is generally ineffective in inducing tumours. Identification of *promoting* agents may thus have more relevance to human carcinogenesis than identification of *initiating* agents. This is another way of saying that the admittedly important and revealing evidence of mutagenicity may not be the most crucial information in relation to carcinogenic risks for man.
3. The exclusion of "complete" carcinogens from man's environment may have only limited scope for cancer prevention, whereas exclusion of independent promoting action may prove to be as important, if not more so.

This presents us with the most challenging problem of all—how to devise practical methods of identifying promoting agents. From what we know at present, it is hard to visualize one single testing system for all potential promoting agents by animal testing, let alone by any short-term technique. A few experimental models for systemic promoting action in animals have been devised in recent years, but these would seem to be too complicated for routine purposes.

I might refer, in this connection, to our own current attempts at transplacental 2-stage carcinogenesis in animals, with a "broad-spectrum" initiator administered to the pregnant mother and the potential promoter to the offspring after birth (Armuth & Berenblum, 1979).

This symposium is perhaps not the appropriate occasion to enlarge on the different methods of cancer prevention, except to refer briefly to the 4 hypothetical points of attack (Berenblum, 1974): (i) by eliminating "complete" carcinogens from the environment (the conventional method of cancer prevention in man); (ii) by eliminating *initiators* from man's environment (based on results of mutagenicity tests); (iii) by eliminating the various *promoting* factors during the long latent period of carcinogenesis (for which no satisfactory testing systems exist at present); and (iv) by *interfering* with the carcinogenic process, as distinct from *eliminating* the incriminating factors (a possibility still at the experimental stage of enquiry (see Wattenberg, 1978) though the results are sufficiently encouraging to offer reasonable prospects of future application).

To conclude, we seem to have come a long way from when the conflict between chemical specificity and chemical diversity of carcinogenic agents dominated our thinking in trying to understand how carcinogens act. The common factor among the diverse agents can now be traced to similarities in *reactivity* of their *metabolic products*, rather than to physico-chemical or structural properties of the *parent compounds*. At a more practical level, epidemiological studies have brought to light a great variety of behavioural, social, dietetic, occupational, and other factors in man's mode of life, which influence his chances of developing cancer (some acting additively, and others apparently as promoting factors); while chemical analysis of air and water pollutants, food contaminants, etc., have added to our knowledge of potential carcinogens in the environment.

Naturally, the more information we can gather about specific carcinogens in our environment and the more we discover about the various kinds of tumour promotion operating in man, the better our chances will be in reducing the prevailing cancer incidence. But in the final analysis carcinogenesis is a biological problem, and only by taking cognizance of the *biological* process, with all its complications, can a rational procedure be formulated in trying to eradicate the disease. Practical applications often call for a pragmatic approach, but a proper understanding of the basic principles is necessary to avoid drawing false conclusions and to minimize the chances of following up false leads.

### REFERENCES

AMES, B. N., MCCANN, J. & YAMASAKI, E. (1975) Methods for detecting carcinogens and mutagens with the Salmonella/mammalian-microsome mutagenicity test. *Mutation Res.*, **31**, 347.

ARMUTH, V. & BERENBLUM, I. (1979) Tritiated thymidine as a broad spectrum initiator in transplacental two-stage carcinogenesis, with phorbol as promoter. *Int. J. Cancer*, **24**, 355.

ASHBY, J. & STYLES, J. A. (1978) Does carcinogenic potency correlate with mutagenic potency in the Ames assay? *Nature*, **271**, 452.

BARTSCH, H. (1976) Predictive value of mutagenicity tests in chemical carcinogenesis. *Mutation Res.*, **38**, 177.

BERENBLUM, I. (1974) *Carcinogenesis as a Biological Problem:* Amsterdam: North-Holland Publ.

BRIDGES, B. A. (1976) Review article: Short term screening tests for carcinogens. *Nature*, **261**, 193.

BROOKES, P. (1975) Minireview: Covalent interaction of carcinogens with DNA. *Life Sciences*, **16**, 331.

DOLL, R. (1977) Strategy for detection of carcinogenic hazards to man. *Nature*, **265**, 589.

GOLDENBERG, L. (ed.) (1974) *Carcinogenesis Testing of Chemicals.* Cleveland, Ohio: CRC Press.

HIGGINSON, J. & MUIR, C. S. (1976) The role of epidemiology in elucidating the importance of environmental factors in human cancer. *Cancer Detection & Prevention*, **1**, 79.

HUEPER, W. C. & CONWAY, W. D. (1964) *Chemical Carcinogenesis and Cancer.* Springfield, Ill.: C. C. Thomas.

IRVING, C. C. (1979) Species and tissue variations in the metabolic activation of aromatic amines. In *Carcinogens: Identification and Mechanisms of Action.* Eds. Griffin & Shaw. New York: Raven Press, p. 211.

MCCANN, J., CHOI, E., YAMASAKI, E. & AMES, B. N. (1975) Detection of carcinogens as mutagens in *Salmonella*-microsome tests: Assay of 300 chemicals. *Proc. Natl Acad. Sci., USA*, **72**, 5135.

MILLER, E. C. (1978) Some current perspectives on chemical carcinogenesis in humans and experimental animals: Presidential address. *Cancer Res.*, **38**, 1479.

PURCHASE, I. F. H., LONGSTAFF, E., ASHBY, J. & 4 others. (1978) An evaluation of 6 short-term tests for detecting organic chemical carcinogens. *Br. J. Cancer*, **37**, 873.

SIVAK, A. (1979) Cocarcinogenesis. *Biochim. Biophys. Acta*, **560**, 67.

SLAGA, *et al.* (Eds.) (1978) Mechanisms of tumor promotion and cocarcinogenesis. In *Carcinogenesis: A Comprehensive Survey.* 2. New York: Raven Press.

STENBÄCK, F., GARCIA, H. & SHUBIK, P. (1974) Present status of the concept of promoting action and cocarcinogenesis in skin. In *The Physiopathology of Cancer.* (3rd edn.) Vol. 1. Basel: S. Karger. p. 155.

VAN DUUREN, B. L. (1976) Tumor-promoting and co-carcinogenic agents in chemical carcinogenesis. In *ACS Monograph* 173*: Chemical Carcinogens.* Ed. C. E. Searle. Washington, D.C.: *Am. Chem. Soc.* p. 24.

WATTENBERG, L. W. (1978) Guest editorial: Inhibition of chemical carcinogenesis. *J. Natl Cancer Inst.*, **60**, 11.

WHO (1964) *Prevention of Cancer.* WHO Technical Report Series, **276**. Geneva.